# Outdoor Air Pollution and Brain Structure and Function From Across Childhood to Young Adulthood: A Methodological Review of Brain MRI Studies

**DOI:** 10.3389/fpubh.2019.00332

**Published:** 2019-12-06

**Authors:** Megan M. Herting, Diana Younan, Claire E. Campbell, Jiu-Chiuan Chen

**Affiliations:** ^1^Department of Preventive Medicine, Keck School of Medicine of University of Southern California, Los Angeles, CA, United States; ^2^Department of Pediatrics, Children's Hospital Los Angeles, Los Angeles, CA, United States; ^3^Department of Neurology, Keck School of Medicine of University of Southern California, Los Angeles, CA, United States

**Keywords:** air pollution, development, brain, neuroimaging, magnetic resonance imaging (MRI)

## Abstract

Outdoor air pollution has been recognized as a novel environmental neurotoxin. Studies have begun to use brain Magnetic Resonance Imaging (MRI) to investigate how air pollution may adversely impact developing brains. A systematic review was conducted to evaluate and synthesize the reported evidence from MRI studies on how early-life exposure to outdoor air pollution affects neurodevelopment. Using PubMed and Web of Knowledge, we conducted a systematic search, followed by structural review of original articles with individual-level exposure data and that met other inclusion criteria. Six studies were identified, each sampled from 3 cohorts of children in Spain, The Netherlands, and the United States. All studies included a one-time assessment of brain MRI when children were 6–12 years old. Air pollutants from traffic and/or regional sources, including polycyclic aromatic hydrocarbons (PAHs), nitrogen dioxide, elemental carbon, particulate matter (<2.5 or <10 μm), and copper, were estimated prenatally (*n* = 1), during childhood (*n* = 3), or both (*n* = 2), using personal monitoring and urinary biomarkers (*n* = 1), air sampling at schools (*n* = 4), or a land-use regression (LUR) modeling based on residences (*n* = 2). Associations between exposure and brain were noted, including: smaller white matter surface area (*n* = 1) and microstructure (*n* = 1); region-specific patterns of cortical thinness (*n* = 1) and smaller volumes and/or less density within the caudate (*n* = 3); altered resting-state functional connectivity (*n* = 2) and brain activity to sensory stimuli (*n* = 1). Preliminary findings suggest that outdoor air pollutants may impact MRI brain structure and function, but limitations highlight that the design of future air pollution-neuroimaging studies needs to incorporate a developmental neurosciences perspective, considering the exposure timing, age of study population, and the most appropriate neurodevelopmental milestones.

## Introduction

There is increasing evidence that air pollutants may disrupt brain and nervous system development in vulnerable populations (1). Given that the brain's structure and function continues to mature well into the third decade of life (2–14), the neurotoxic effects of outdoor air pollution may be present throughout the entire dynamic neurodevelopmental process, as a result of exposure from childhood, through adolescence, and into young adulthood (15–17). Extant data on neurodevelopmental effects of air pollution suggest that both prenatal and postnatal exposure is associated with deficits in intelligence quotient (IQ) (18–22), as well as a broad range of cognitive domains (21, 23, 24), subclinical mental health problems (25, 26) as well as risk for Autism Spectrum Disorders (27–29). Together, these findings suggest outdoor air pollution exposure may impact cognitive development and emotional behaviors, yet questions remain regarding the mechanisms influencing the structural and functional brain alterations that may underlie these associations observed in children and adolescents. The aim of this study was to systematically review the current air pollution epidemiologic literature that used Magnetic Resonance Imaging (MRI) techniques to study brain structure and function in children and adolescents. While recent reports have highlighted this growing area of research (30, 31), the current review not only offers an updated state-of-the-science summary and synthesis from the developmental neuroscience perspectives, but also provides a thorough methodological critique of the literature that was largely missed in earlier reviews.

## Methods

We conducted an extensive search of the MEDLINE database through PubMed using a combination search of a list of “air pollution” terms and a list of “brain imaging” terms. A comprehensive strategy for a combination search was developed to identify articles published before September 6, 2018 and with keyword components including air pollution and functional and/or structural MRI, using both MESH terms and Title/Abstract keywords and excluding animal studies and studies published in non-English languages. The search algorithm was as follows: [(“magnetic resonance image”[title/abstract] OR “magnetic resonance images”[title/abstract] OR “magnetic resonance imaging”[title/abstract] OR “MRI” [Title/Abstract] OR “white matter hyperintensity”[title/abstract] OR “white matter hyperintensities”[title/abstract] OR “neuroimage”[title/abstract] OR “neuroimages”[title/abstract] OR “neuroimaging”[title/abstract] OR “neuroinflammation”[title/abstract] OR “systemic inflammation”[title/abstract] OR “white matter volume”[title/abstract] OR “white matter volumes”[title/abstract] OR “brain structure”[title/abstract] OR “brain volume”[title/abstract] OR “brain volumes”[title/abstract] OR “neurotoxic”[Title/Abstract] OR “neurotoxicity”[Title/Abstract] OR “neurotoxicities”[Title/Abstract] OR “functional connectivity”[Title/Abstract] OR “Brain/pathology”[mesh] OR “Brain/physiopathology”[Majr] OR “Magnetic Resonance Imaging”[Mesh] OR “Cognition Disorders/pathology”[Mesh] OR “Cognition Disorders/chemically induced”[Majr] OR “White Matter/pathology”[Majr]) AND (“Air Pollution”[Title/Abstract] OR “Air Pollutant”[Title/Abstract] OR “Air Pollutants”[Title/Abstract] OR “Particulate Matter”[Title/Abstract] OR “Ozone”[Title/Abstract] OR “Nitrogen dioxide”[Title/Abstract] OR “Nitrogen oxides”[Title/Abstract] OR “Sulfur Dioxide”[Title/Abstract] OR “black carbon”[title/abstract] OR “elemental carbon”[title/abstract] OR “Vehicle Emission”[Title/Abstract] OR “Vehicle Emissions”[Title/Abstract] OR “diesel”[Title/Abstract] OR “diesel exhaust”[Title/Abstract] OR “diesel exhausts”[Title/Abstract] OR “vehicle exhaust”[Title/Abstract] OR “vehicle exhausts”[Title/Abstract] OR “vehicular exhaust”[Title/Abstract] OR “vehicular exhausts”[Title/Abstract] OR “road traffic”[Title/Abstract] OR “PM2.5”[Title/Abstract] OR “PM10”[Title/Abstract] OR “coarse particle”[Title/Abstract] OR “coarse particles”[Title/Abstract] OR “ultrafine particle”[Title/Abstract] OR “ultrafine particles”[Title/Abstract] OR “Polycyclic aromatic hydrocarbon”[Title/Abstract] OR “Polycyclic aromatic hydrocarbons”[Title/Abstract] OR “Air Pollution”[Mesh] OR “Particulate Matter”[Mesh] OR “Ozone”[Mesh] OR “Nitrogen dioxide”[Mesh] OR “Nitrogen oxides”[Mesh] OR “Sulfur Dioxide”[Mesh] OR “Vehicle Emissions”[Mesh] OR “Air Pollution/adverse effects”[Majr] OR “Polycyclic Aromatic Hydrocarbons/adverse effects”[Mesh] OR “Polycyclic Aromatic Hydrocarbons/poisoning”[Mesh] OR “Polycyclic Aromatic Hydrocarbons/toxicity”[Mesh] OR “Inhalation Exposure/adverse effects”[Mesh])]. For resulting articles, we obtained their abstracts, plus full texts if needed, to determine each publication's relevance to our review. The selection process to identify relevant papers for a full review consisted of two phases of screening, including a title and abstract screening, followed by a full-text screening. Two reviewers conducted the two phases independently (C.C. and D.Y.), and in case of doubt, a third reviewer (M.H.) was consulted. When selected for full-text screening, additional inclusionary criteria were met to be reviewed in detail. Inclusionary criteria for this review were as follows: (1) full-length original research article; (2) reporting individual-level data on exposures to outdoor air pollution, (3) at least one of the MRI outcome measures was examined (e.g., structural T1, diffusion-weighted T2, echo planar imaging T2, etc.); and (4) studying children, adolescents, or young adults (aged ≤ 30 years) at the time of MRI scans. Studying exposure effects during young adulthood is relevant to the scope of our review because the brain continues to develop into the third decade of life (4, 7–9, 11–14, 32, 33). Studies were excluded if they met the following criteria: (1) not an original paper; (2) indoor or occupational air pollution; (3) no MRI outcome of interest; (4) experimental studies; (5) human studies without individual-level exposure data; and (6) studies conducted in adults aged >30 years. For all the identified relevant original and review articles, we also looked into the title/abstract of listed bibliographies to identify additional articles that may not have been captured through our initial search of the MEDLINE database. The same extensive search process was repeated using the ISI Web of Knowledge dataset.

## Results

**Figure 1:**
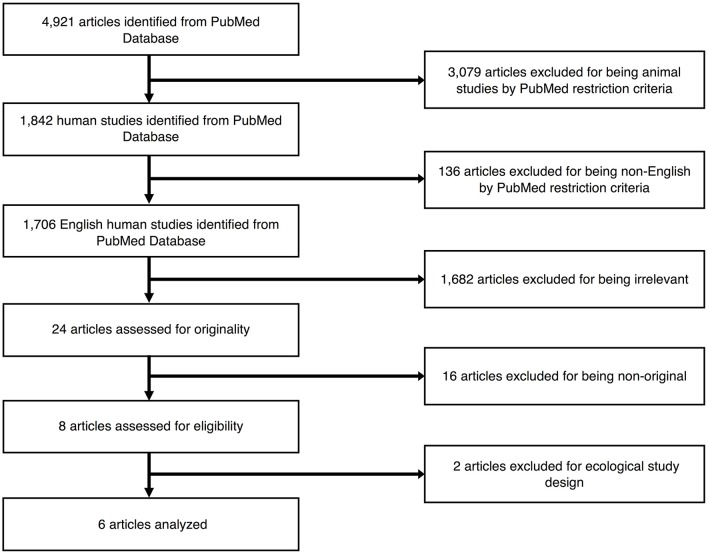
Selection process of the articles to be reviewed.

Figure 1 outlines the study selection process. Using the comprehensive search strategy, 4,921 unique articles were identified from PubMed, with 1,842 of them meeting the criteria of including humans. One hundred thirty-six articles were then removed for being published in a language other than English. Of the remaining 1,706 studies, 1,682 articles were removed following title and abstract screening, as the articles did not meet the following criteria outlined above (i.e., exclusion of individual-level data on exposures to outdoor air pollution or MRI outcome measures; participants were adults or elderly subjects at the time of MRI scans). The remaining 24 articles were selected for a full-text review, with 16 articles ineligible as they did not meet the criteria of being an original research article (i.e., review articles or commentaries). The remaining 6 articles were considered relevant original research articles and included in the final review. No additional studies were identified from our ISI Web of Knowledge database or through the manual search of listed bibliographies of relevant articles. Details of the 6 studies are presented in Table 1. All studies included only cross-sectional MRI data from pre-adolescents (<13 years old), with no studies conducted during mid-to-late adolescence (13–19 years) or young adulthood (20–30 years old). Participant samples were subsets from 3 larger children cohorts: (1) the Brain Development and Air Pollution Ultrafine Particles in School Children (BREATHE) project with traffic as the primary source of air pollution in Barcelona, Spain (*n* = 4); (2) the Generation R Study in Rotterdam, The Netherlands where air pollution is generated from traffic, industrial, and other point sources (*n* = 1); and (3) the Columbia Center for Children's Environmental Health (CCCEH) cohort with traffic as the primary outdoor source of intra-urban air pollution in New York City (*n* = 1). These studies employed various approaches to exposure assessment. The CCCEH cohort used personal air samples and urinary biomarkers to estimate prenatal and post-natal exposures to polycyclic aromatic hydrocarbons (PAHs), respectively. In the BREATHE project, investigators directly measured particulate matter with aerodynamic diameter of <2.5 μm (PM_2.5_) and nitrogen dioxide (NO_2_), in both the school courtyard (outdoor) and in the classrooms (indoor) during two 1-week campaigns for the warm and cold seasons, as well as residential NO_2_ and PM_2.5_ at the time of the study and the prenatal period. The Generation R study used residential location data during pregnancy and applied several validated land-use regression (LUR) models to estimate particulate matter with aerodynamic diameter of <10 μm (PM_10_), PM_2.5_, NO_2_, and absorbance of fine particles as a proxy of elemental carbon (EC) during the entire fetal period, accounting for changes in home address during pregnancy. All included studies reviewed were commendable in using either a strategic participant sampling approach to reduce other pollutant exposures and/or adjusting for second-hand smoke (i.e., a source of indoor pollution) and socioeconomic factors. However, tactics for reducing additional confounds, including socioeconomic status, parental or child's education, and other social adversities in the residential or school neighborhood, were diverse (see Table 1 for details). Below, we synthesize these details and the reported findings on the exposure-outcome associations, according to the primary exposure time window under study.

**Table 1:** Summary studies.

**References**	**Study design and MRI sample**	**Exposure**	**Outcome**	**Main findings**	**Confounders adjusted/controlled**	**Conclusions**
**OUTDOOR AIR POLLUTION AND BRAIN IMAGING**						
Peterson et al. (34)	40 right-handed children born to nonsmoking Dominican and African-American women 18–35 years old residing in Washington Heights, Harlem, or the South Bronx in NYC were recruited 1998–2006 through local prenatal care clinics and followed *in utero* to 7–9 years of age	Personal air monitors to measure 8 airborne PAHs (benz[a]anthracene, chrysene, benzo[b]fluroanthene, benzo[k]fluroanthene, benzo[a]pyrene, indeno[1,2,3-cd]pyrene, disbenz[a,h]anthracene, and benzo[g,h,i]perylene) and determine maternal exposures over 48 h period during 3rd trimester, with mean ± standard deviation (SD) of 5.13 ± 6.20 ng/m^3^. Spot urine samples collected from child at age 5 used to measure postnatal urinary PAH metabolites, with *N* = 38 and mean ± SD of 14999.4 ± 19795.8 ng/L. Source of pollutants: traffic emissions	3T GE, with 8 channel head-coil; T1 weighted: TR = 4.7 ms, TE = 1.3 ms, acceleration factor = 2, 256 × 256 matrix, 160 slices, 1 mm thickness; ANALYZE 8.0 and in-house software; structural MRI's, surface mapping. Correction using False Discovery Rate (*p* < 0.05).	PAH correlated inversely with morphological measures in frontal, superior, temporal, parietal, and rostrocaudal extent of the mesial surface mostly in the Left hemisphere; underlying white matter mainly driving effect. Pearson correlation coefficients and 95% confidence intervals: Prenatal PAH levels: Left lateral superior prefrontal cortex: −0.57 (−0.75, −0.304); Left superior temporal gyrus: −0.51 (−0.71, −0.23); Left precuneus: −0.50 (−0.71, −0.22); Left medial prefrontal cortex: −0.56 (−0.75, −0.30) Postnatal (age 5) PAH levels: Left dorsal-lateral prefrontal cortex: −0.47 (−0.69, −0.18); Left medial dorsal prefrontal cortex: −0.52 (−0.72, −0.25)	Child's age and sex; prenatal cotinine levels, measures of postnatal PAH exposure at age 5 years, handedness	Prenatal PAH relates to less left hemisphere white matter; while postnatal PAH contributes to less white matter in dorsal prefrontal regions
Pujol et al. (35)	263 children (mean age 9.7 ± 0.9 years old; range: 8–12.1 years) from the BREATHE project who were selected from 39 schools in Barcelona (schools selected based on modeled NO_2_ values) and had been in the school for at least 18 months	Pollutant levels in school courtyards were sampled twice during 1-week periods (8 h/day) separated by 6 months in the warm (year 2012) and cold (year 2012/2013) periods. Elemental carbon (measured in PM_2.5_) was measured with High-Volume samplers and NO_2_ with passive dosimeters (96 h period), with mean ± SD of 0.92 ± 0.30 μg/m^3^ and range of 0.42–1.92 μg/m^3^. A single traffic-related pollutant indicator was computed using the weighted average of these two, using the following formula:	1.5T GE with 8 channel head-coil; T1 weighted: TR = 11.9 ms, TE = 4.2 ms, Flip = 15°, 256 × 256 matrix, 134 slices, 1.2 mm thickness; SPM (version 8) VBM and FREESURFER (version not reported). DTI: 25 directions with *b*-value = 1,000 s/mm^2^, TR = 8,300 ms, TE = 94 ms, Flip = 90°, 128 × 128 matrix, 26 slices, 5 mm thickness with no gap, spin-echo single-shot EPI; FMRIB FDT, accounted for head motion.	No significant association was identified between air pollution and any anatomical, DTI or metabolic brain measurement. Traffic-related air pollution was significantly associated with: (1) weaker functional connectivity between regions belonging to the DMN (i.e., between the medial frontal cortex and the angular gyrus bilaterally); (2) stronger functional connectivity between the medial frontal cortex seed region and the frontal operculum at the lateral boundary of the DMN; (3) lower deactivations (rest > task map) during passive viewing and listening in the supplementary motor area and somatosensory cortex.	Age, sex, academic achievement, difficulties scores, obesity, parental education, home and school vulnerability index, distance from home to school and public/non-public school category	Traffic-related air pollution was associated with functional brain changes, but there was no relationship with brain anatomy, white matter microstructure, or membrane metabolism
		[(EC/group median) + (NO_2_/group median)/2] Source of pollutants: traffic emissions	RS-FMRI (eyes closed): TR = 2,000 ms, TE = 50 ms, Flip = 90°, 64 × 64 matrix, 22 slices, 4 mm with 1.5 mm gap, 180 EPI volume; SPM8 preprocessing with 8 mm Gaussian filter; high-pass filter (~0.008 Hz), white matter, CSF, and global signal removal; scrubbed for motion; Functional connectivity maps using 3.5 mm spheres of frontal lobe (*n* = 4) and caudate (*n* = 2) seeds; Correction with Monte Carlo and a family-wise error rate (*p* < 0.05) and Bonferroni per connectivity map (*n* = 4). Sensory FMRI task: TR = 2,000 ms, TE = 50 ms, Flip = 90°, 64 × 64 matrix, 22 slices, 4 mm with 1.5 mm gap, 120 EPI volume; block design (ABABABAB) of 30 s fixation and 30 s of visual-auditory input; Correction with Monte Carlo and a family-wise error rate (*p* < 0.05). Proton (1H) Spectroscopy: PROBE-SV and STEAM sequence, TR = 2000 ms, TE = 30 ms, 128 signal averages, voxel = 23 × 14 × 14 mm in the left frontal white matter; NAA FWHM 0.09 ppm and creatine RMS >8.	*T*-values (non-adjusted vs. adjusted by age and sex): RS-FMRI Medial frontal seed map: Left Lateral frontal cortex (+ correlation): 3.6 vs. 4.1; Left Parietal cortex (– correlation): 3.5 vs. 3.2; Right Parietal cortex (– correlation): 3.5 vs. 3.2 RS-FMRI Dorsal frontal seed map: Left Parietal cortex (+ correlation): 3.6 vs. 3.5; Right Lateral frontal cortex (– correlation): 3.7 vs. 3.5; Right Insula (– correlation): 4.3 vs. 4.3 RS-FMRI Posterior cingulate seed map: Left Lateral frontal cortex (+ correlation): 3.3 vs. 3.2 RS-FMRI Supplementary motor area seed map: Left Prefrontal cortex (+ correlation): 4.4 vs. 4.6; Right Prefrontal cortex (+ correlation): 3.5 vs. 3.6; Left Parietal cortex (+ correlation): 3.3 vs. 3.4; Right Parietal cortex (+ correlation): 4.1 vs. 3.9; Left Anterior cingulate cortex (– correlation): 3.6 vs. 3.7 Sensory FMRI task: Right Somatosensory cortex (+ correlation): 3.7 vs. 3.8; Left Premotor cortex (+ correlation): 3.7 vs. 3.6		
Pujol et al. (36) Brain and Behavior	263 children (mean age 9.7 ± 0.9 years old; range: 8–12.1 years) from the BREATHE project who were selected from 39 schools in Barcelona (schools selected based on modeled NO_2_ values) and had been in the school for at least 18 months	Copper (measured in PM_2.5_) levels in school courtyards were sampled with High-Volume samplers twice during 1-week periods (8 h/day) separated by 6 months in the warm (year 2012) and cold (year 2012/2013) periods and analyzed using ICP-MS, and then averaged to obtain the yearly exposure levels, with mean ± SD of 8.7 ± 3.0 ng/m^3^ and range of 3.7–13.8 ng/m^3^. Source of pollutants: traffic emissions, industrial sources, and railway	1.5T GE with 8 channel head-coil; T1 weighted: TR = 11.9 ms, TE = 4.2 ms, Flip = 15°, 256 × 256 matrix, 134 slices, 1.2 mm thickness; SPM (version 8) VBM (5 FWHM) and FREESURFER (version not reported). DTI: 25 directions with *b*-value = 1,000 s/mm^2^, TR = 8,300 ms, TE = 94 ms, Flip = 90°, 128 × 128 matrix, 26 slices, 5 mm thickness with no gap, spin-echo single-shot EPI; FMRIB FDT, accounted for head motion. RS-FMRI (eyes closed): TR = 2,000 ms, TE = 50 ms, Flip = 90°, 64 × 64 matrix, 22 slices, 4 mm with 1.5 mm gap, 180 EPI volume; SPM8 preprocessing with 8 mm Gaussian filter; high-pass filter (~0.008 Hz), white matter, CSF, and global signal removal; scrubbed for motion; Functional connectivity maps using 3.5 mm spheres of frontal lobe (*n* = 4) and caudate (*n* = 2) seeds; Correction with Monte Carlo and a family-wise error rate (*p* < 0.05).	Higher copper levels were associated with: (1) higher gray matter concentration in the striatum, specifically in the caudate nucleus, with no effect on tissue volume; (2) higher FA in white matter close to the caudate nucleus and in the caudate nucleus; (3) changes in the complex architecture of neural tissue diffusion; (4) reciprocal reduction of functional connectivity between the caudate nucleus and the frontal lobe operculum (bilaterally). Beta coefficients and 95% confidence intervals: Gray matter density: Left caudate nucleus: β = 0.3 (0.1, 0.5) DTI Fractional Anisotrophy (FA): Left caudate nucleus: β = 0.1 (0.05, 0.2) RS-FMRI: Left frontal cortex to Left caudate: β = −0.1 (−0.2, −0.1)	Age, sex, academic achievement, academic difficulties score, obesity, parental education, home and school vulnerability index, public/nonpublic school category, socioeconomic status, elemental carbon, other toxic agents (Pb, Mn, Sb, and Fe)	Association between environmental copper exposure in children and alterations of caudate structure and function
Mortamais et al. (37)	242 children (median age 9.7 years; range: 8–12 years) from the BREATHE project who were selected from 35 of 39 schools in Barcelona (schools selected based on modeled NO_2_ values) and on average at school for 6.5 years before study; removed 19 participants b/c parents said smoked at home	Pollutant levels in school courtyards were sampled twice during 1-week periods (8 h/day) separated by 6 months: Jan to June 2012 and Sept 2012 to Feb 2013. PAHs (measured in PM_2.5_) was measured with High-Volume samplers. Outdoor and indoor PAHs: (benz[a]anthracene (BAAN), chrysene (CHRYS), benzo[b+j+k]fluroanthene (BFL), benzo[e]pyrene (BEP), benzo[a]pyrene (BAP), indeno[1,2,3-c,d]pyrene (IP), and benzo[g,h,i]perylene (BGP)) levels were obtained by averaging the two one-week measures, which were seasonalized, with BAP mean ± SD of 99 ± 62 pg/m^3^ and range of 20–304 pg/m^3^, and a PAHs mean ± SD of 1,458 ± 704 pg/m^3^ and range of 597–3,235 pg/m^3^. Weekly-averaged NO_2_ concentrations with passive dosimeters were also obtained. Source of pollutants: traffic emissions	1.5T GE with 8 channel head-coil; T1 weighted: TR = 11.9 ms, TE = 4.2 ms, Flip = 15°, 256 × 256 matrix, 134 slices, 1.2 mm thickness; FREESURFER volumes (version not reported). No correction for multiple comparisons.	No associations with brain parenchymal fraction, putamen, or globus pallidus volumes. Higher outdoor school PAHs was associated with caudate volumes. Beta coefficients and 95% confidence intervals per IQR increase: Caudate nucleus, mm^3^: Total PAHs: β = −132.9 (−245.0, −20.8); *p* = 0.020; BAP: β = −150.6 (−259.1, −42.1); *p* = 0.007 Boys (n = 123): Total PAHs: β = −19.2 (−211.0, 172.6); *p* = 0.844; BAP: β = −48.6 (−219.8, 122.5); *p* = 0.577 Girls (n = 119): Total PAHs: β = −192.3 (−364.1, −20.5); *p* = 0.028; BAP: β = −212.0 (−382.8, −41.2); *p* = 0.015 No significant interactions between sex and PAHs observed for brain volumes Null findings: Brain parenchymal fraction, %: Total PAHs: β = 0.3 (−0.2, 0.7); *p* = 0.240; BAP: β = 0.0 (−0.4, 0.5); *p* = 0.929. Putamen, mm^3^: Total PAHs: β = 26.4 (−128.3, 181.2); *p* = 0.738; BAP: β = 13.0 (−139.2, 165.2); *p* = 0.867; Globus pallidus, mm^3^: Total PAHs: β = 4.5 (−46.1, 55.2); *p* = 0.861; BAP: β = −16.9 (−64.9, 31.0); *p* = 0.488	Age, sex, ICV, maternal education, home socioeconomic vulnerability index, residential exposure to NO_2_ and PM_2.5_, classroom noise	Exposure to PAHs associated with subclinical changes on the caudate
Guxens et al. (38)	783 children (6–10 years old) from the Generation R Study, a population-based birth cohort in Rotterdam, The Netherlands, who were born between April 2002 and Jan 2006. Oversampled based on certain maternal exposures during pregnancy (i.e., cannabis, nicotine, selective serotonin reuptake inhibitors, depressive symptoms, and plasma folate levels) and child behavior problems (i.e., ADHD, pervasive developmental problems, dysregulation problems, and aggressive problems)	Air pollution monitoring campaigns took place over three 2-week periods for NO_2_ in 80 sites and PM_10_, PM_2.5_, and PM _absorbance_ (a proxy for elemental carbon) in 40 sites in 2009 to 2010 across The Netherlands and Belgium. Coarse PM calculated as the difference between PM_10_ and PM_2.5_. The 3 measurements were averaged, adjusting for temporal variation. LUR models were then used to estimate air pollution exposure levels at mothers' geocoded residential address during the entire fetal period. Background monitoring network sites (*n* = 7) were used to back-extrapolate to the fetal period, accounting for changes of home address during pregnancy. a range of 16.8–28.1 μg/m^3^, the coarse (PM_10_) median value was 11.8 μg/m^3^ with a range of 9.2–17.8 μg/m^3^, the proxy for elemental carbon (PM _absorbance_) median value was 1.9 × 10^−5^ m^−1^ with a range of 1.2–3.6 10^−5^ m^−1^. Source of pollutants: intra-ubran road traffic, traffic on highways, and industrial and other point sources	3T GE with 8 channel head coil; T1 weighted: TR = 10.3 ms, TE = 4.2 ms, Flip = 16°, 0.9 × 0.9 mm in-plane resolution, 186 slices, 0.9 mm thickness; FREESURFER (version 5.1) volumes and cortical thickness. Corrected using Monte-Carlo null-Z w/10,000 iterations *(p* < 0.1).	No associations were seen between air pollution exposure during fetal life and global brain volume measures at 6–10 years of age. Significant associations between air pollution exposure during fetal life and regional cortical thickness at 6–10 years of age. Fully adjusted beta coefficients and 95% confidence intervals representing the differences in thickness (mm) per each increase of 5 mg/m^3^ of PM_2.5_, 5 mg/m^3^ of PM_10_, and 10^−5^ m^−1^ of PM_absorbance_: PM_2.5_: Right precuneus region: β = −0.045 (−0.062, −0.028); *p* < 0.001; Right Pars opercularis region: β = −0.024 (−0.033, −0.014); *p* < 0.001; Pars orbitalis region, right: β = −0.028(−0.043, −0.012);*p* = 0.001; Right rostral middle frontal region: β = −0.029 (−0.041, −0.018); *p* < 0.001; Right superior frontal region: β = −0.029 (−0.043, −0.016); *p* < 0.001; Left cuneus region: β = 0.022 (−0.035, −0.009); *p* = 0.002 PM_10_: Right Lateral orbitofrontal region: β = −0.037 (−0.059, −0.016); *p* = 0.001 PM_*ab*__sorbance_: Left fusiform region: β = −0.105 (−0.160, −0.049); *p* < 0.001 Null findings: NO_2_: Total brain volume: β = 124 (−1118, 1,375); *p* = 0.84; Cortical gray matter volume: β = −60 (−853, 733); *p* = 0.88; Cortical white matter volume: β = 199 (−287, 685); *p* = 0.42; Subcortical gray matter volume: β = 36 (−17, 89); *p* = 0.18; Ventricular volume: β = 4 (−57, 64); *p* = 0.90 PM_2.5_: Total brain volume: β = −3079 (−7790, 1,632); *p* = 0.20; Cortical gray matter volume: β = −2598 (−5583, 387); *p* = 0.09; Cortical white matter volume: β = −268 (−2096, 1,559); *p* = 0.77; Subcortical gray matter volume: β = −60 (−258, 138); *p* = 0.55; Ventricular volume: β = −96 (−323, 131); *p* = 0.40; PM_10_: Total brain volume: β = −4868 (−10337, 822); *p* = 0.09; Cortical gray matter volume: β = −3542 (−7059, 8); *p* = 0.05; Cortical white matter volume: β = −1129 (−3215, 1127); *p* = 0.34; Subcortical gray matter volume: β = −92 (−325,148); *p* = 0.46; Ventricular volume: β = −100 (−372, 168); *p* = 0.45; PM_absorbance_: Total brain volume: β = −2861 (−18745, 24,467); *p* = 0.79; Cortical gray matter volume: β = −2683 (−16377, 11,012); *p* = 0.70; Cortical white matter volume: β = 5,807 (−2566, 14,180); *p* = 0.17; Subcortical gray matter volume: β = 418 (−497, 1,334); *p* = 0.36; Ventricular volume: β = −64 (−1108, 979); *p* = 0.90	Parental educational levels, monthly household income, parental countries of birth, parental ages, maternal prenatal smoking, maternal prenatal alcohol use, maternal parity, family status, and maternal psychological distress, calculated pre-pregnancy BMI, maternal IQ when children were 6; child's gender, age at scanning, genetic ancestry	Children exposed to higher PM levels during fetal life had thinner cortex in several brain regions of both hemispheres
Alemany et al. (39)	163 children (mean age 9.3 ± 0.82 years old; range: 8–12.1 years) from the BREATHE project who had genetic data available. Children were selected from 38 schools in Barcelona and 1 adjacent city, Sant Cugat del Vallés (schools selected based on modeled NO_2_ values) and had been in the school for at least 18 months. 22.7% (*n* = 37) were APOE-e4 carriers	Pollutant levels in school courtyards were sampled twice during 1-week periods (8 h/day) separated by 6 months: Jan to June 2012 and Sept 2012 to Feb 2013. PAHs (measured in PM_2.5_) was measured with High-Volume samplers. Outdoor and indoor PAHs: (benz[a]anthracene (BAAN), chrysene (CHRYS), benzo[b+j+k]fluroanthene (BFL), benzo[e]pyrene (BEP), benzo[a]pyrene (BAP), indeno[1,2,3-c,d]pyrene (IP), and benzo[g,h,i]perylene (BGP)) levels were obtained by averaging the two one-week measures, which were seasonalized, with PAHs mean ± SD of 1546.29 ± 775.08 pg/m^3^. Elemental carbon (measured in PM_2.5_) was measured with High-Volume samplers and NO_2_ with passive dosimeters (96 h period), with NO_2_ mean ± SD of 47.74 ± 12.95 μg/m^3^. Source of pollutants: traffic emissions	1.5T GE with 8 channel head-coil; T1 weighted: TR = 11.9 ms, TE = 4.2 ms, Flip = 15°, 256 × 256 matrix, 134 slices, 1.2 mm thickness; FREESURFER (version 5.3). No correction for multiple comparisons.	Beta coefficients and 95% confidence intervals of basal ganglia volumes by annual average of air pollution exposure to PAHs, EC, and NO_2_ (per IQR increase): Caudate nucleus, mm^3^: Total PAHs: β = −120.1 (−211.2, −29); EC: β = −110 (−250.5, 30.6); NO_2_: β = −265.1 (−474.3, −56) Putamen, mm^3^: Total PAHs: β = 9.1 (−115.2, 133.5); EC: β = −15.2 (204.7, 235.1); NO_2_: β = 93.4 (−364.3, 177.5) Globus pallidus, mm^3^: Total PAHs: β = 1.1 (−37.2, 39.5); EC: β = −22.2 (−101.6, 57.1); NO_2_: β = −35.6 (−133.9, 62.7) In e4 carriers and non-carriers: Caudate nucleus, mm^3^: Total PAHs: e4 carrier β = −590.2 (−1032.3, −148.15) vs. non-carrier β = −137.1 (−322.0, 47.84); *p* = 0.04; EC: e4 carrier β = −375.0 (−776.81, 26.88) vs. non-carrier β = −49.8 (−236.6, 137.1); *p* = 0.11; NO_2_: e4 carrier β = −737.9 (−1201.3, −274.5) vs. non-carrier β = −157.6 (−388.8, 73.6); *p* = 0.03 Putamen, mm^3^: Total PAHs: e4 carrier β = −13.6 (−272.5, −245.3) vs. non-carrier β = 19.45 (−456.4, 495.3); *p* = 0.90; EC: e4 carrier β = −31.0 (−444.3, 382.25) vs. non-carrier β = 52.9 (−206.3, 312.1); *p* = 0.55; NO_2_: e4 carrier β = −63.75 (−586.3, 458.8); non-carrier β = −82.4 (−405.4, 240.5); *p* = 0.74 Globus pallidus, mm^3^: Total PAHs: e4 carrier β = −43.1 (−212.9, 126.75)vs. non-carrier β = −5.1 (−81.0, 70.88); *p* = 0.40; EC: e4 carrier β = −13.1 (−166.3, 140.2) vs. non-carrier β = −26.10 (−102.0, 49.8); *p* = 0.97; NO_2_: e4 carrier β = −49.6 (−237.9, 138.6) vs. non-carrier β = −44.15 (−138.7, 50.35); *p* = 0.79	Age, sex, ICV, maternal education, maternal smoking, exposure to tobacco at home, home socioeconomic vulnerability index, residential exposure to NO_2_ and PM_2.5._	Association between annual average air pollution exposure to PAHs and NO_2_ and smaller caudate volumes was larger in children carrying the APOE-e4 allele compared to non-carriers

### Prenatal Exposure

**Figure 2:**
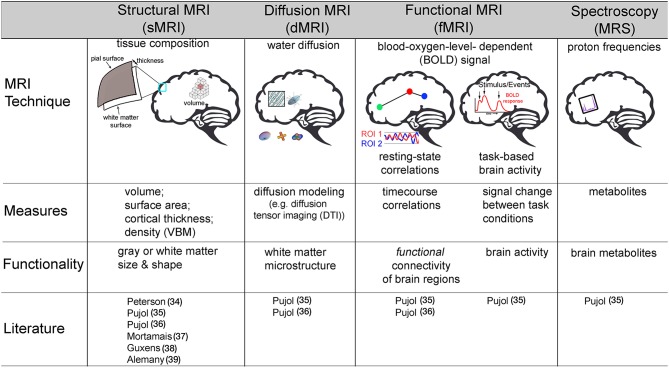
Description of various non-invasive magnetic resonance imaging (MRI) techniques used to assess how outdoor air pollution exposure relates to brain structure and function in children. VBM, voxel based morphometry; DTI, diffusion tensor imaging; ROI, region of interest. Pujol (35); Pujol (36).

Both prenatal exposure studies utilized structural MRI, which is a technique that allows for the quantification of tissue types and morphological metrics of the brain, including gray matter (i.e., cell bodies) and white matter (i.e., myelination of axons) volume (size in mm^3^), surface area (mm^2^), density, and cortical thickness (i.e., mm, distance between gray/white matter boundary and pia matter) (Figure 2). Using structural MRI, Peterson et al. was the first study to examine the association between outdoor air pollution and brain structure in 40 children (7–9 years) selected from a larger African-American and Dominican birth cohort study in New York City from 1998 to 2006 (34). Of the 727 mother-newborn pairs in the CCCEH cohort, 40 children were identified to capture a full range of prenatal PAHs exposure measured by personal monitoring for 48 h in the 3rd trimester. The results suggested that white matter, comprised of myelination, or the insulation of axons (i.e., connections) between brain cells, was most affected. Higher levels of prenatal PAHs exposure were associated with reductions in white matter surface area and were largely located on the left side of the brain. After adjusting for prenatal exposure, urinary level of PAH metabolites measured at age 5 were also found to be associated with smaller white matter surface area in a specific region of both the right and left side of the frontal lobe, known as the dorsolateral prefrontal cortex. Correlations were assessed between PAHs (prenatal and postnatal) and various behavioral scores measured by The Wechsler Intelligence Scale for Children (IQ and processing speed) and Child Behavior Checklist (Anxiety-Depression, Attention, DSM-ADHD, DSM-Conduct, and Externalizing scores) at age 7 years. Of the fourteen correlations, only prenatal PAH and processing speed were found to be significant (*p* < 0.05, uncorrected). Using a Sobel test, white matter surface area partially mediated an association between higher prenatal PAHs exposure and slower processing speed. A key strength of this study was a strategic sampling approach of the children in order to minimize confounding factors of other ambient chemicals (e.g., second-hand smoke, insecticides). In addition, in MRI studies where the brain is segmented into multiple regions or into smaller units, up to 500,000 or more statistical tests are commonplace. For this reason, multiple comparison corrections are an imperative step in reducing Type I error in MRI brain research (40, 41). Importantly, Peterson and colleagues' results were corrected accordingly; adding an important level of rigor to its methodology. However, this study was also not without limitations. In addition to the small sample size, the current study only included age and sex of the child, and did not account for family socioeconomic status or other social factors as covariates in the fully adjusted models. While the children were all selected based on a minority population, it is unclear if socioeconomic factors even within this strategically chosen sample may be confounding results. Nonetheless, this pioneering study provided evidence that prenatal and postnatal PAH exposure in particulate matter may impact a child's brain development.

In a large (*n* = 783) group of school-age children (ages 6–10 years) based on the Generation R study, investigators examined prenatal exposure and total brain volumes and thickness of gray matter in the cortex (38). Based on an air pollution monitoring campaign of NO_2_ across 80 sites and PM across 40 sites in The Netherlands and Belgium in 2009–2010, LUR models were developed, and exposures to NO_2_, coarse particles (PM_10_ minus PM_2.5_), PM_2.5_, and a proxy for EC were assigned to maternal residential locations. Investigators found no association between prenatal exposure and overall brain size (e.g., total whole brain, total gray, total white, total subcortical gray, and total ventricular volume). However, greater prenatal PM_2.5_ exposure was associated with reduced cortical thickness in portions of the frontal lobe, as well as the parietal and occipital lobes. Higher levels of coarse particle and absorbance of fine particles (e.g., marker for EC) exposure during the fetal period were associated with thinner gray matter in separate frontal and occipital regions of the cortex, including the lateral orbitofrontal and fusiform regions, respectively. Causal mediation analyses were then employed using bootstrapping to determine whether cortical thickness is a mediator of the association between the pollutant and behavioral performance on a set of cognitive and emotional tasks. Adjusted associations were determined between air pollutants and behavioral performance based on the cognitive function of each identified regions. Of the 18 statistical tests performed, only PM_2.5_ was found to significantly relate to inhibition errors on the response set task (*p* = 0.02). Of the 5 regions associated with PM_2.5_, inhibition errors on this test was found to only relate to thickness in one region in the frontal lobe (i.e., rostral middle frontal; *p* = 0.02) and another region in the parietal lobe (i.e., precuneus; *p* = 0.05). Formal causal mediation analyses showed that thickness in these brain regions partially mediated the relationship between prenatal fine particle exposure and poor inhibitory control. The strengths of the current study were the large sample size and the number of social factors adjusted for in their final models, including family socioeconomic status (i.e., parental education, household income) as well as parental and other prenatal factors (i.e., parental age, maternal smoking, and other drug use, etc., maternal stress, maternal IQ). In addition, the authors performed a thorough adjustment for multiple comparisons for their MRI outcomes. A limitation to the study, however, is that the initial design of the Generation R study was to oversample on both maternal exposures (i.e., drug, alcohol, psychiatric medication) during pregnancy as well as child behavior problems. Thus, the generalizability of the current study may be limited by its potential sampling bias.

In the two prenatal studies, to date, the interpretations are also limited in that they did not account for the exposure measures temporally proximate to the MRI scans or other brain health outcomes. Specifically, these studies have not characterized, or accounted for, the exposure of the individual spanning the various years prior to assessing brain development. For example, Peterson and colleagues measured one 48-h period of exposure during the third trimester period and a single urine sample at age 5. Using two relatively abbreviated periods of exposure to assign prenatal and postnatal exposure is likely not accurate in reflecting both the amount and variability of exposure of a given individual prior to measuring the outcome. Similarly, Guxens and colleagues use of LUR of exposure were limited to the prenatal period, without exposure information for over 6–10 years leading up to the MRI visit. As such, it is difficult to rule out potential air pollution effects during the postnatal period in which the brain continues to develop. The following section on childhood exposure further highlights the potential importance of postnatal exposure on MRI related brain outcomes.

### Childhood Exposure

Pujol et al. was the first to examine how childhood air pollution exposure relates to brain MRI-measured structure and function. Using various types of MRI neuroimaging, the investigators included 263 children (ages 8–12 years) recruited from the BREATHE project in Barcelona, Spain. High-volume active samplers for PM_2.5_ and passive dosimeters for NO_2_ were placed both inside classrooms and outside in the school courtyard during a 1–week (8 h/day) air monitoring campaign, which was conducted twice (one in warm season and the other in cold season) during the study period. An MRI assessment, including structural MRI, diffusion MRI (dMRI), resting-state functional MRI (rs-fMRI), Magnetic Resonance Spectroscopy (MRS), and task-based fMRI, was collected 1 year after measuring air pollution exposure at school. The investigators calculated traffic-related pollution scores using EC and NO_2_ (35). Given that EC and NO_2_ are highly correlated and have been found to be related to vehicle exhaust in Barcelona, the authors calculated a traffic related pollutant indicator using the weighted average of exposure to EC and NO_2_. This traffic-related pollution index was associated with differences in brain function as measured by fMRI. FMRI measures the blood oxygen level-dependent (BOLD) signal (42) as an estimate of neural activity. The BOLD signal capitalizes on the tight coupling of blood flow and oxygenation when local neurons are activated. By mapping changes in the BOLD signal, fMRI can indirectly measure neuronal activity across time while individuals rest, known as resting-state fMRI (rs-fMRI) (43), or in relation to completing some sort of activity or task, often referred to as task fMRI (42) (Figure 2). Using both of these types of fMRI, Pujol and colleagues, found that higher traffic-related pollution was related to brain activity during rest as well as during a sensory fMRI task (35). Specifically, higher traffic air pollution exposure was associated with less functional connectivity (e.g., BOLD signal correlations) between the frontal to parietal lobes (i.e., medial frontal to parietal cortex), between two frontal lobe regions (i.e., dorsal frontal cortex to lateral frontal and insula), and between motor and frontal regions (i.e., supplementary motor area to anterior cingulate) while subjects were at rest. Also while at rest, higher traffic-related exposure was also linked to stronger brain activity between motor areas and both the right and left side of the prefrontal and parietal lobes, as well as the distinct frontal lobe regions with the parietal lobe and cingulate cortex (i.e., medial frontal to lateral frontal, dorsal frontal to parietal, and posterior cingulate to lateral frontal cortex). An entire field of literature has focused on understanding common patterns of brain activity while individuals rest, which are known as functional connectivity networks (43, 44). Pujol and colleagues noted that altered patterns of functional activity with traffic pollution included some brain regions that are part of the resting state default mode network (DMN) whereas other regions are part of a functional connectivity networks known to be involved in more goal-directed behaviors, like the frontal-parietal (FP) network. Thus, the authors highlighted that traffic-related air pollution may lead to poor brain activity *within these specific networks* as well as impairments in brain activity *between these different networks* in children (35). The map of brain activity seen in the medial frontal lobe during rest was also found to relate to motor reaction time (collected from a cognitive battery performed with the children at school) (35). This study also found that greater air pollution from vehicle exhaust was linked to less deactivation in the somatosensory and premotor brain regions when individuals were presented with both visual and auditory stimuli in the MRI machine. Interestingly, no associations were found between the traffic-exposure score and other MRI outcomes collected, including brain volume or cortical thickness (sMRI), membrane metabolism (MRS), or white matter microstructure (via dMRI).

Using the data collected from the same cohort of children from the BREATE project, copper was measured in PM_2.5_ and found to correlate with road traffic, industrial activity, and in some schools, train proximity. Higher average 1-year exposure to copper was associated with greater gray matter density, but not volume, of both the right and left caudate as well as increased gray matter thickness in the left supplementary motor area (36). In addition, this study also found copper to relate to white matter microstructure using the technique dMRI (Figure 2). DMRI is an MRI techniques that provides additional insight on water diffusion to assess microstructural properties of various tissues (32). More uniform restriction, quantified with dMRI using a term known as “fractional anisotropy” (FA), is thought to reflect more myelination in white matter regions, as well as axon organization, and/or axon size. Using this technique, higher copper exposure was related to higher values of FA in the caudate nucleus and its adjacent white matter tract (36). It is important to note that in the field of developmental neuroscience, higher values of FA in children are thought to reflect more myelination (e.g., insulation of the cell connections) and bigger axons, which typically occur with healthy, normative brain development (9, 32). Therefore, an association between higher copper exposure and “more mature” biomarkers of white matter development is rather counterintuitive. The authors postulate that this unexpected relationship may be due to a difference in the number of connections that may cross in the region; which is a known methodological limitation of this method (45). Unfortunately, the authors did not report other common diffusion metrics (e.g., axial or radial diffusivity) or estimate individual white matter tracts for all participants in the study; which are necessary to help clarify these unexpected findings between copper and white matter microstructure. Lastly, higher copper exposure in the year preceding neuroimaging collection correlated with reduced functional connectivity (e.g., negative correlations) for the caudate nucleus to portions of the frontal lobe (i.e., fronto-operculum) and the supplementary motor area to a portion of the parietal lobe, known as the supramarginal gyrus. However, again there were also increased connectivity between regions of the frontal lobe (i.e., medial prefrontal cortex to fronto-operculum) and regions of the frontal lobe (i.e., medial prefrontal cortex) to auditory cortex (36). Although a negative association was also found between copper exposure and reaction time variability on an attention network task in this MRI sample, the authors did not report whether additional analyses were performed to examine the relation among exposure to airborne copper, behavior, and brain-MRI measures.

More recently the BREATHE project has also examined how PAHs, EC, and NO_2_ relate to basal ganglia volumes using structural MRI (37, 39). A negative association was found between outdoor benzo[a]pyrene (BAP) and both the right and left caudate nucleus volumes; a similar finding was found when using a total score of outdoor PAHs (37). Neither BAP nor total PAH levels related to the ratio of total gray and white matter to intracranial volume (i.e., overall brain size) (37). Expanding on these findings, 163 children with both MRI and genetic information were examined to determine if vulnerability to air pollution may vary by the e4 allele in the apolipoprotein E (APOE) gene – a primary genetic risk factor for Alzheimer's disease (39). Higher average annual NO_2_ and PAH were again shown to relate to smaller caudate volumes from the BREATHE study, with a larger effect estimate in children carrying the APOE-e4 allele (39). In both studies, no associations were found between exposure levels (PAHs, EC, or NO_2_) and other nearby regions, such as the putamen or globus pallidus volumes. Adjusting for residential-based NO_2_ and PM_2.5_ exposure (assigned via LUR) at the time of the study (37, 39) was not found to change the results.

Important in helping reduce the likelihood of spurious associations, the BREATHE project considered a number of confounding factors in their fully-adjusted models. For example, family socioeconomic status was accounted for by maternal education, whereas a vulnerability index (e.g., education, unemployment, and occupation by census tract) was assessed to account for potential neighborhood socioeconomic effects (35–37, 39); although the use of these variables seemed to vary by study reports. In addition, classroom noise was measured in one study; albeit the data were not shown and inclusion as a covariate during model testing was unclear (37). In terms of limitations, only two of the four studies from the BREATHE project corrected for multiple comparisons (see Table 1 for details). Although there is less concern about the proximity between exposure and outcomes, the BREATHE project also faces the challenge of accounting for potential effects of exposure at other periods of the child's development. Of the studies to date, only one of the recently published BREATHE studies accounted for prenatal exposure based on residential LUR estimates (37). In fact, regardless of examining the prenatal or postnatal period of exposure, no air pollution-MRI study to date has fully characterized the potential temporal exposures across the life span of the child and their various impacts on brain maturation. For example, each study focused on just a handful of exposures and quantified them over abbreviated periods of maturation. Together, these methodological challenges of understanding both the temporal dynamics of exposure as well as characterization of various air pollutants may result in inaccurate prediction of exposure, which suggests existing studies may suffer from information bias (e.g., misclassification of exposure). Moving forward a more thorough assessment of various exposures across development are necessary to strengthen the conclusions that can be drawn regarding the timing of exposure and potential impacts on neurodevelopment.

## Discussion

Our systematic review identified 6 studies on outdoor air pollution that reported associations between outdoor air pollution and white matter (e.g., surface area and microstructure), cortical and subcortical gray matter (e.g., cortical thickness, volumes), as well as brain function (e.g., resting state and task-based brain activity patterns). These findings also suggest that outdoor air pollution exposure may harm neurocognitive maturation through its impact on brain structure, with partial mediations being reported in Peterson et al. (34) and Guxens et al. (38). When considering the type and timing of exposure, various social confounders, as well as the different MRI outcomes under study, these findings are far from conclusive. For instance, the two prenatal exposure studies found opposite effects on the type of brain tissue impacted. Peterson et al. (34) found significant associations between PAH and white matter surface area (but not with gray matter), whereas in a larger sample Guxens et al. (38) found associations with particles (EC, PM_10_, PM_2.5_) and gray matter thickness, but not white matter measures. These two studies employed different methodological MRI preprocessing and analytic techniques, making it difficult to directly compare or amalgamate their imaging results. For example, cortical thickness, surface area, and gray matter volume capture distinct neurobiological phenotypes and these outcomes vary as a function of development across childhood and adolescence (46). In contrast, the reported associations with postnatal exposures were much more similar; albeit conducted by a single research group. In the BREATHE project, 1-year average exposures to traffic-related air pollutants (e.g., copper, PAHs, TRAP [EC+NO_2_], and NO_2_) was associated with smaller caudate volume (35, 37, 39), altered functional brain activity to sensory stimuli and altered patterns of intrinsic connectivity of large-scale functional networks (35, 36). However, these postnatal exposure studies to date are based solely on the BREATHE project; the generalizability and the reproducibility of their novel findings need to be examined in other studies of different population context. These and other considerations need to be addressed in future research.

### Recommendations for Future Research

#### Incorporation of a Developmental Neuroscience Perspective

**Figure 3:**
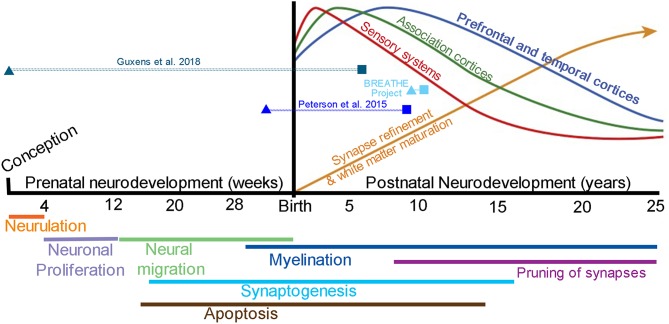
Brain development from conception to young adulthood. Stages of brain development during the prenatal period (weeks) thru postnatal period of childhood and adolescence (years). Top: Postnatal quadratic trajectories of gray matter volume patterns seen by MRI, with sensory systems (red) peaking earlier in development, followed by association (green) and prefrontal and temporal (blue) cortices; a continual increase in white matter volume is seen well into the 3rd decade of life (yellow); Adapted from Nelson et al. (47) with permission from National Academies Press. The timing of outdoor air pollution (triangles) and the age of MRI assessment (squares) are presented for each of the included studies [note BREATHE project includes (35–37, 39)]. Bottom: Molecular and cellular processes that contribute to patterns of brain maturation as seen by MRI, which may be vulnerable to outdoor air pollution from fetal development to young adulthood; Adapted from Tau and Peterson (48) with permission from Springer Nature. Neurulation, folding of the neural plate into the neural tube; Neuronal proliferation, formation of two major brain cell types known as neurons and glial cells; Neural migration, movement of neurons to their final destination in the brain; Apoptosis, planned cell death; Synaptogenesis, creation of synapses allowing for communication via neurotransmitters between neurons; Myelination, formation of a myelin sheath to allow for faster cell conduction (e.g., myelination is often termed “white matter” via MRI); Pruning of synapses, removal of connections between cells (i.e., axon or dendrite) allowing for improvements in network capacity and efficiency.

Brain development is hallmarked by an orchestra of events that span both the prenatal and postnatal period of development (Figure 3). Longitudinal studies of child and adolescent brain development using MRI provide strong evidence that brain development is dynamic and continual, and that individual differences exist in brain structure and function at the beginning of an MRI study and change over time (i.e., intercepts and slopes) (49). Postnatal neurodevelopmental trajectories vary by brain region, with sensory, motor, and language reaching peak volumes in early childhood and the prefrontal cortex, hippocampus, amygdala, and cerebral white matter volumes continuing to mature across adolescence and into the mid-to-late twenties (4, 7, 9, 10, 32, 50). Moreover, new cell growth and plasticity of cell connections are especially apparent in the hippocampus and amygdala across the lifespan, and are required for adult cognitive and emotional-based learning and memory processes.

Evidence from the reviewed studies suggests that exposure to ambient air pollution during both the prenatal (34, 38) and childhood periods (34–37, 39) are associated with MRI brain outcomes in children under age 12. Two studies found adverse effects of postnatal estimates in statistical models that adjusted for prenatal exposure (34, 37), providing evidence for the independent effects of postnatal exposure periods and highlighting the importance of studying the continuing impact of neurotoxic air pollutants on the extended developmental trajectories beyond school age. However, the studies that included postnatal periods of exposure focused primarily on the pre-adolescent period and no study examined how outdoor air pollution exposure may impact the dynamic periods of adolescent and young adult brain maturation despite known differences in adolescent and young adult brain structure and function as compared to children. Future research needs to be conducted in adolescents and young adults in order to better understand how air pollution may impact the unique neural systems throughout the entire developmental process of brain maturation. Since trajectories of growth vary by brain region, one might expect that early exposure (prenatal and/or early childhood) may impact early developing systems such as sensory, motor, and language systems, while executive functions and self-regulation of emotion and reward processes may remain vulnerable to neurotoxic insults during the teenage years and as individuals transition into young adulthood. Although the literature remains scant, the observation of air pollution exposure during childhood and its effect on caudate volumes may illustrate the importance of exposure time window. Specifically, prenatal studies did not detect this association (34, 38), whereas associations of average annual BAP exposure and bilateral caudate nucleus volumes was seen in children after adjusting for prenatal exposure (37). Moreover, growth of the caudate has been shown to continue from childhood to young adulthood (4, 51), and together, with the prefrontal cortex, portions of the caudate play a vital role in various executive functions, including cognitive control (52). Therefore, the design of future air pollution-neuroimaging studies needs to incorporate a developmental neurosciences perspective, considering the exposure timing, age of study population, and the most appropriate neurodevelopmental milestones, regardless of whether the study is focused on the neurotoxic effects in the period of fetal, childhood, adolescent, or young adult development. Studies interested in exposure later in neurodevelopment will also have the challenge of addressing how earlier periods of exposure may influence their results, as questions also remain if early neurotoxic effects persist or become exaggerated across the lifespan.

#### Longitudinal Design With Repeated Exposure Assessment and MRI Scans

Existing studies included only one-time assessment of structural morphometry and functional measures by brain MRI scans. Similarly, previous studies have been limited by not being able to fully characterize the exposure histories across different time windows relevant to the neurodevelopmental stages. For instance, previous studies on the prenatal exposure and developing brains rarely account for the recent exposures in close temporal proximity to MRI measures. Studies with more comprehensive exposure characterization over time as well as more than one MRI assessment can help determine whether ambient air pollution may change the “starting points” (i.e., intercepts) and/or may change the trajectory (i.e., slopes) of brain maturation as well as offer a more accurate understanding of the amount and timing of exposure that may be most detrimental. For example, longer durations of exposure to ambient air pollution may presumably result in more severe and/or permanent effects on brain structure and function. If true, rather than assessing a single time window of exposure such as the prenatal window and/or adjusting for more recent exposure during childhood, larger effects may be seen if we examine how annual and/or cumulative child or adolescent exposure is associated with trajectories of neurodevelopment. Therefore, longitudinal studies with more comprehensive exposure characterization and repeated MRI scans are needed to enhance the validity of the study findings to date, as well as fully characterize the specific neurodevelopmental trajectories affected by outdoor air pollution exposure during vulnerable windows across both the prenatal and postnatal periods.

#### Confounders and Covariates

Studies to date have used different approaches in addressing the presence of other potential confounders, including socioeconomic status, parental or child's education, social adversities in the residential or school neighborhood, and related spatial confounders. Further studies should consider following the lead of Guxens et al. (38) who implemented a directed acyclic graph approach based on existent literature. All studies to date have adjusted for age, sex, and intracranial volume (i.e., overall differences in head size when the dependent variable of interest was brain volume) (see Table 1 for details), but other known confounders are likely to include the participant's family- and neighborhood-level socioeconomic background (e.g., family income, maternal education, neighborhood poverty) and various lifestyle factors (e.g., physical activities). This is especially important in places like the United States since exposures are higher among minorities (53), as well as for people with less than a high-school education compared to those with a high-school or college education, and among persons with income below the poverty line (54). Depending on sources, air pollution levels can be different in urban vs. rural environments, suggesting spatial covariates may also confound the exposure effect estimates if known to affect neurobehavioral development, such as traffic noise and greenspace. Similarly, neurodevelopment covariates to explore may include prenatal factors (e.g., maternal age, pre-term birth) and other physical health (e.g., body mass index (BMI), pubertal maturation, medications). Future studies need to clearly present the methods by which they measured and determined to include or exclude confounding factors in fully adjusted models. This will strengthen the results of future studies to mitigate concerns about differences in social class and race as well as other physical environmental factors that may otherwise lead to potential confounding bias.

#### Different Sources and Exposure Characteristics

Further research is needed to improve our understanding of the specificity and source(s) of exposure, as well as other characteristics most harmful to the developing brain. The reported primary pollutants included PM_2.5_, PM_10_, NO_2_, or various PM_2.5_ constituents (e.g., PAHs, copper, and EC). Most studies alluded to traffic as being the primary source of exposure, although not clearly documented in all studies. To reduce the potential confounding by other known environmental neurotoxins, Peterson et al. (34) recruited an informative sample with no or very low levels of prenatal exposures to second-hand smoke, lead, and chlorpyrifos. Others performed statistical adjustment in their data analyses to control for the potential influences by other co-pollutants or conducted sensitivity analyses regarding second-hand smoke exposure (36–39). One other study dealt with correlated pollutants by creating a composite score of exposure, and therefore did not adjust for multiple pollutants (35). Discrepant findings are likely to occur if future studies do not consider a multitude of sources and various exposures that children may encounter within and between various studies. Even when studies both assess the same criterion pollutant, such as PM_2.5_, the chemical composition may vary between geographical locations (55). By assessing and accounting for various exposures in the same sample, stronger conclusions can be made to strengthen policy implications regarding which exact types of exposure are most harmful. For example, while all the studies investigated derivatives of particle pollution, these studies did not quantify various other air pollutants, such as airborne gases, carbon monoxide, sulfur oxides, or other volatile organic compounds. In addition, the exposure methods of the previous studies varied from personal monitoring, stationary monitoring, and LUR prediction modeling. Advantages and disadvantages of various types of exposure assessments for outdoor air pollution exposure, including LUR, urinary biomarkers, and personal monitoring sensors, have been reported in detail by recently focused reviews (56, 57). Detailed critiques of these approaches were beyond the scope of this review, but we note that the appropriate choice of exposure assessment methods depends on both the study design and research questions. For instance, studies that aim to retrospectively capture long-term exposure on neurodevelopmental processes in a well-characterized existing cohort may find that spatiotemporal models are useful to gather information about exposure over days, months, and years; especially given recent advances in establishing more fine grain spatiotemporal (i.e., 1 × 1 km grid, daily estimate) predictions for various particle and gaseous pollutants (58–61). For studies built on ongoing or newly launched cohorts, it will be desirable to collect information on individual-level behaviors of the child, such as the amount of time and patterns of activities for playing outdoors or time spent in other microenvironments (e.g., in vehicles; in classrooms), as such data may be used to determine the exposure sources, refine the specific characteristics of exposure, and reduce the exposure measurement error. On the other hand, although personal sampling allows for the most complete assessment of personal exposure across various microenvironments, technical limitations, such as participant compliance and burden have made this method more difficult for large scaled, population-based studies (57). For these same reasons, personal sampling is usually limited to a very brief period of time (i.e., 24 or 48 h), leading to a rather crude estimate of possible exposure to try and associate with neural processes of brain development that occur over months and years when captured with MRI. This latter point is especially of importance, as it remains to be elucidated if acute time-varying factors, such as exposure the day or week before, would impact various MRI outcomes. For example, reliability differences in MRI estimates within the same individuals (62) may suggest macrostructural changes detected by MRI may be better suited for detecting effects of chronic exposure, whereas functional metrics may be more sensitive to acute conditions. Future studies should consider these strengths and weaknesses of each technique depending on their specific question(s) in relation to how air pollution may impact the brain during development.

#### Assessing the Heterogeneity of Exposure Effect

The putative developmental neurotoxic effects of exposure to ambient air pollution may vary by demographic features and genetic factors (63). Of the included studies, two studies examined sex differences in the effect, with one suggesting larger effects in girls (34) and the other reporting no difference between the sexes (37). Given the sex differences seen in the associations between air pollution exposure and cognition, with some studies reporting greater impairments in males compared to females (21, 22, 24, 64, 65), future studies should examine sex as a potential modifier and report summary statistics by sex. Similarly, outdoor air pollution exposure is associated with various adverse health effects in children (66), such as pre-term birth (67, 68), increased BMI (69), increased asthma risk (70), delayed lung function growth (70, 71), and vitamin-D deficiency (72, 73). These clinical attributes may modify the impact exposure has on brain structure and function, and future studies need to examine their potential contributions to the heterogeneity in the neurotoxicity of exposure to ambient air pollution. In addition to a confounder, SES may also be an effect modifier. While Peterson et al. (34) was based on the larger CCCEH cohort of minority pregnant women of low SES backgrounds; none of the MRI studies to date included a large and diverse enough sample to examine SES as a modifier. This is likely an important issue to address, as previous cognitive studies in adolescents and young adults have found that the adverse effects of exposure can be exponentially larger in low SES families (22). Thus, more research is needed to examine the possible social disparities in exposure effects on MRI brain outcomes. Lastly, only one study examined how genetics influenced the association between air pollution and brain structure (39). These findings of an interaction between genotype and exposure (39) corroborate animal studies (74), further highlighting the importance of genetic differences in how neurodevelopment may be affected by air pollution. Genes involved in oxidative stress and inflammatory pathways are logical candidates for future studies as well as functional polymorphisms that are involved in neuronal proliferation and differentiation (e.g., brain-derived neurotrophic factor) (75) and neurotransmitter transporters and receptors (e.g., 5-HTTLPR) (76). Large-scale studies with adequate statistical power will be needed to examine whether individuals may be differentially affected by early life exposure to outdoor air pollutants based on additional environmental and biological factors.

#### Advanced Multi-Modal Neuroimaging

Several MRI techniques were used based on 1.5-Tesla (*n* = 4) or 3-Tesla (*n* = 2) MRI with an 8-channel head-coil, including structural MRI (CCCEH, BREATHE, and Generation R), functional MRI (BREATHE), diffusion MRI (BREATHE), and magnetic resonance spectroscopy (MRS) (BREATHE). Figure 2 illustrates key concepts about each of these non-invasive MRI techniques. Future studies have a number of advancements in hardware and sequence development in the field of neuroimaging at their disposal, such as 3T and 7T scanners combined with a 32- or 64-channel radiofrequency head-coil (instead of a 1.5 or 3T with an 8-channel head-coil), which improves the MRI signal-to-noise ratio. Similarly, sequences with higher spatial resolution for diffusion MRI and fMRI scans are readily available with voxel sizes around 1.5–2 mm^3^ as compared to 3–5 mm^3^ a few years ago. Like the BREATHE project, studies should aim to perform various advanced MRI modalities and examine multiple metrics per imaging modality within the same population to allow for a more complete characterization of brain structure and function.

##### Regional-specific findings with structural MRI

Despite previous studies linking vehicle related traffic to smaller head circumferences during gestation (77), MRI findings in children suggests air pollution exposure does not lead to global differences in whole brain volume or intracranial size (35–38). The contrasting findings between head circumferences and total brain size could be due to a number of reasons. First, head circumference measurements were taken by ultrasound and only examined the exposure and outcome variables during pregnancy. The included studies have examined both prenatal and postnatal windows of exposure, but all brain MRIs outcomes were measured during childhood (6–12 years). Furthermore, while head circumference and brain volume have been found to correlate at very young ages (<6 years), at older ages (7–42 years) head size is no longer an accurate proxy for brain volume (78). Thus, brain MRI may provide a much more precise measure of brain development, especially given the dynamic structural changes seen across childhood and adolescence. Recent studies also suggest that head size and intracranial volume may be strongly linked to genetics (79), which may explain why no associations exist between air pollution exposure and intracranial volume or whole brain metrics, even in the largest MRI sample studied to date (38).

Rather than global volume impairments, regional specificity in the existing studies suggest some neural processes may be more vulnerable to the neurotoxic effects of air pollution, including the prefrontal cortex, white matter, and caudate. Depending on data preprocessing, structural MRI can segment gray matter (primarily composed of cell bodies and synapses) and white matter (primarily myelination of axons) into volume estimates based on regions of interest (ROI) (reported in BREATHE, Generation R), assess density with voxel based morphometry (VBM) (as reported in BREATHE), cortical thickness (reported in BREATHE and Generation R), and/or surface area (as reported in CCCEH). These structural outcomes are not synonymous, and each morphometric (e.g., thickness, volume, surface area) has been found to follow a different developmental trajectory (7, 46). Therefore, a more granular targeted analysis in terms of both regional-specificity as well as examination of each morphometric outcome will help to elucidate which brain regions and neural processes may be most vulnerable to the neurotoxic effects of air pollutants.

##### Task FMRI and rs-FMRI

Of the reviewed studies, only a single fMRI task was utilized to measure BOLD response to sensory stimuli. We found no studies that utilized task-based fMRI to examine the neural substrates underlying the reported deficits in various cognitive (e.g., working memory or inhibitory control) (80) or emotional (e.g., anxiety or aggression) behaviors that have been linked with outdoor air pollution exposure in children and adolescents (21, 23–26). Air pollution MRI studies using working memory, attention, inhibitory control, and emotion fMRI tasks are warranted. In terms of resting state, only four seed regions were previously used to examine functional connectivity of regions included in the DMN and the fronto-parietal networks (35). However, it is unclear if exposure affects other neural networks, including the somatomotor, visual, cingulo-opercular, or dorsal attention, which are also known to develop across childhood into young adulthood (81–84). Moreover, future studies combining resting state fMRI and graph theory analyses (44) will be able to more directly test if air pollution exposure results in maintaining strong *within-network* connectivity and poor *between-network* connectivity, ultimately reflecting altered maturation of resting state networks. Given that the BREATHE project was the only study to implement these techniques, fMRI remains an untapped resource for elucidating how outdoor air pollution affects brain activity across development.

##### Structural white matter connectivity

Multi-shell diffusion-weighted imaging may provide an unparalleled opportunity to further our understanding of how air pollution impacts white matter maturation. Sequences using High Angular Resolution Diffusion Imaging (HARDI) sequences (i.e., higher number of gradient directions) allows for more complex models of water dispersion to more accurately perform tractography to assess white matter fiber bundles (85). Similarly, multi-shell dMRI acquisition (e.g., including more than one b-value) can allow for *neurite orientation dispersion and density imaging* (NODDI) analyses (86). This allows for calculation of orientation dispersion (ODI) and neurite density (NDI) indices, of which increases in ODI may best reflect myelination (87), whereas reduced NDI may reflect geometrical complexity of neurite architecture in the human central nervous system (88). Utilization of these novel techniques in future studies may improve our ability to further elucidate our understanding of how outdoor air pollution impacts white matter. For example, previous associations between air pollution and white matter surface area (34) and FA (36) suggested that myelination (i.e., the lipid and protein sheath along axons that allows for faster signal conduction) and/or oligodendrocytes (i.e., myelin forming glial cells) may be affected. Moreover, by comparing the reported findings of copper and white matter FA values (36) with a well-known white matter tract atlas (89), these affected white matter regions can be further categorized into the superior corona radiation (SCR), anterior corona radiation (ACR), internal capsule (IC), or genu of the corpus callosum (gCC). Employing multi-shell and NODDI techniques may elucidate the degree by which outdoor air pollution affects the development of specific white matter fiber bundles and myelination along those fiber tracts. Thus, studies using these novel dMRI techniques have an unparalleled opportunity to further assess how air pollutants may impact the structural integrity and myelination of these white matter tracts as they develop across childhood, adolescence, and well into the third decade of life (32, 90).

##### Cerebral blood flow

It remains unknown if outdoor air pollution leads to alterations in neurovasculature. Despite known effects on cardiovascular health (91), including increased blood pressure and hypertension (92, 93) and neurovasculature inflammation (94), no study has examined if outdoor air pollution may alter cerebral perfusion. A common MRI technique that can complement structural MRI and fMRI is arterial spin labeling (ASL). ASL measures the delivery rate of blood flow to brain tissue, termed cerebral blood flow (CBF) (95). CBF is regulated by astrocytes (e.g., neuronal support cells), based on the demands of neuron activity (which utilizes oxygen and glucose), and is thus variable across the brain. Thus, measuring CBF can be a useful technique to clarify if fMRI results are confounded by changes in the hemodynamic response, but also may elucidate additional mechanisms—neurovasculature and astrocytes (given their vital role in regulating CBF)—by which outdoor air pollution may affect the developing brain.

#### A Translational Approach Toward Understanding Mechanisms

While the exact mechanisms remain unknown, it is thought ambient air pollution may impact the brain *in utero* or during postnatal development either via systemic and/or direct effects. During the prenatal period, possible mechanisms include nanoparticles (smaller than 240 nm) bypassing the placenta to impact the developing fetus (96) and/or maternal changes in the immune system (97, 98), as indicated by changes in cord blood immune system seen to occur with air pollution exposure (99). Postnatal exposure may also affect the brain by direct neurotoxicity and/or acting secondarily through systemic changes (1, 100). Fine and coarse PM can deposit into airways and lung tissue (101). Trace metals, endotoxins, and other smaller soluble compounds can then interact with tissues to produce reactive oxygen species or induce signaling cascades that increase inflammation. Following an increase in inflammation, inflammatory markers enter the circulatory system and disrupt the blood brain barrier, allowing pro-inflammatory cytokines, and other substances to enter the brain. In addition to systemic effects, it has been hypothesized that metals and other toxins, including ultrafine particles, may take a more direct route to the brain through the olfactory system (102, 103), although support for this hypothesis is mixed. Animal models continue to show that PM exposure leads to various changes within the brain, including neuroinflammation, oxidative stress, microglial activation, neurovascular damage, altered neurotransmitters, and up-regulation of genes encoding inflammatory cytokine pathways (100, 104–108). These experimental findings were supported by several neuropathological reports suggesting activation of neuroinflammatory markers and microglia, impairments in vasoconstriction, and lesions in the frontal lobe in the brain autopsies of children, young adults, and animals living in highly polluted cities (109, 110).

A limitation of human MRI is that it is unable to decipher cellular and molecular level changes that may occur as a result of outdoor air pollution. Thus, in studying the fundamental questions of how air pollution affects these brain systems, future research should aim to perform a translational approach that bi-directionally informs researchers performing both human and animal studies. For example, MRI studies will be vital to elucidate which neural networks are especially vulnerable to the neurotoxic effects of air pollutants in humans. These findings can then inform animal exposure studies to elucidate the key cellular and molecular changes. In the same realm, existing animal studies already have shown additional key brain regions that are impacted by exposure, including lateral ventricle size and the hippocampus (65, 111, 112). These animal studies provide insight into the need to take a targeted approach to consider examining the volumes of these two structures, but also the function and connectivity of the hippocampus with larger networks. These are just a few examples, yet the possibility of using a translational research approach will ultimately aid us in understanding the mechanisms underlying how outdoor air pollution exposure impacts neurodevelopment.

## Conclusions

In summary, prenatal, and postnatal exposure to chemicals and particulate matter from outdoor sources, including the chemical components indicative of exposures to traffic-related air pollution, may lead to alterations in specific neural networks. Longitudinal studies with multi-modal, advanced MRI measures are needed to determine how the timing of exposure from conception to young adulthood influences brain structure and function. Examining the potential heterogeneity by which outdoor air pollution impacts both regional specificity as well as large-scaled neural networks will greatly advance our understanding of air pollution neurotoxicology in populations and contribute to the critical knowledge base needed to inform both prevention strategies and public health policy.

## Data Availability Statement

The raw data supporting the conclusions of this manuscript will be made available by the authors, without undue reservation, to any qualified researcher.

## Author Contributions

MH made substantial contributions to the conception of this review, analysis, interpretation of the data, and writing the manuscript. DY and CC were involved with the acquisition, analysis, and interpretation of the data as well as revising the manuscript for intellectual content. J-CC was involved in the conceptualization of the project as well as the interpretation of the data and revising of the manuscript. All authors gave final approval of the version to be published and agreed to be accountable for all aspects of their respective contributions of the work.

### Conflict of Interest

The authors declare that the research was conducted in the absence of any commercial or financial relationships that could be construed as a potential conflict of interest.
